# The impact of temperature and sediment resuspension on microbial eukaryote recruitment from shallow Baltic Sea sediment

**DOI:** 10.3389/fmicb.2025.1654505

**Published:** 2025-11-21

**Authors:** Ingrid Sassenhagen, Daniel P. R. Herlemann, Kaja Gentsch, Anke Kremp, Matthias Labrenz, Jörg Dutz

**Affiliations:** Department of Biological Oceanography, Leibniz Institute for Baltic Sea Research, Warnemünde, Germany

**Keywords:** sediment resuspension, eukaryotic microbial community composition, resting stages, germination, metabarcoding, shallow water

## Abstract

Germination from plankton resting stages is often inhibited by burial and anoxic conditions in the sediment. Resuspension of surface sediments by storms might, therefore, represent an important mechanism that facilitates germination. We investigated the impact of sediment resuspension on the composition of planktonic microbial eukaryote community, assessed through 18S rRNA gene metabarcoding, by incubating sediment cores from Greifswald Bay (Baltic Sea) either undisturbed or with regular resuspension events for 4 months at rising temperatures. In this experiment, the planktonic eukaryotic community composition was mostly characterized by temporal species succession likely driven by the gradual increase in water temperature. Furthermore, NO_3_^−^ concentrations and sediment resuspension were identified as important factors shaping the eukaryotic community composition. Nitrate concentrations were significantly higher in the control than in the mixed sediment cores, as resuspension likely caused a significant loss of nitrate to the sediment. Unexpectedly, eukaryotic alpha diversity was not significantly altered by mixing, but the community composition changed due to the germination of previously buried species. These results highlight the importance of sediment resuspension for the activation of buried resting stages and for altering nutrient concentrations in the water column impacting planktonic microbial community composition.

## 1 Introduction

Many microbial eukaryotic species form resting stages to survive unfavorable conditions (Ellegaard and Ribeiro, 2017) and establish seed banks in the sediment. The recruitment of planktonic eukaryotes from the sediment has a strong impact on community composition in the water column, as germinating cells can serve as an inoculum for a new bloom. Germination in many microbial eukaryotes is often regulated by seasonal stimuli, such as temperature and photoperiod, and an internal clock (Eilertsen et al., 1995; Perez et al., 1998; Kremp and Anderson, 2000). However, the greatest densities of resting stages often occur well below the sediment–water interface (Anderson et al., 1982; Keafer et al., 1992; Nehring, 1994). This burial in the sediment and the associated dark and anoxic conditions that frequently occur in the Baltic Sea inhibit germination of resting stages (Nehring, 1996; Rengefors and Anderson, 1998; Kremp and Anderson, 2000). These dormant cells can, therefore, only be considered part of the pool of potential recruits to the plankton if mechanisms that return these cells to the oxygenated sediment surface or into the water column exist. Resuspension by storms and bioturbation by benthic animals can fulfill this important function (Nehring, 1996; Ståhl-Delbanco and Hansson, 2002; Rengefors et al., 2004). Another effect of sediment mixing is that nutrients, which are important for microbial growth, can be released to the water and may potentially stimulate the growth of germinating cells. Gibson et al. (2000), for instance, found that the internal cycling of silica, a prerequisite for diatom growth, was enhanced by bioturbation. Although ecological drivers for germination have individually been investigated in several microbial eukaryotes, the impact of sediment resuspension on natural eukaryotic communities in shallow coastal areas has rarely been studied and is therefore poorly understood.

Greifswald Bay, with 510 km^2^, is the largest estuary at the German Baltic Sea coast and represents a natural, shallow basin with an average water depth of 5.8 m and a maximum depth of 13.5 m in the eastern part (Kanstinger et al., 2018). The lagoon has two connections to the Baltic Sea, a large opening with a shallow sill in the east and a narrow channel on the western side (Strelasund). Hydrodynamics are primarily governed by wind. Mixing results in a well-oxygenated water column to the sea-bed, and wind-driven advection exchanges water masses between the Bay and the Baltic Sea (Munkes, 2005). Higher nutrient supplies from urban and agricultural development in the catchment of Greifswald Bay between 1950 and 1990 have led to a phase shift from a macrophyte-dominated to a phytoplankton-dominated shallow water ecosystem (Schiewer, 2001). Due to the limited water exchange with the Baltic Sea, resting stages of various microorganisms are likely retained in Greifswald Bay (Bauer et al., 2013) and accumulate as seed banks in the sediment.

It is currently unknown how sediment resuspension affects different species in natural microbial communities and the seasonal species succession. On the one hand, resuspension might change species diversity in the water column. Diversity could increase when cells from different sediment layers are able to germinate. However, resuspended cells might also quickly establish an abundant population in the water column and outcompete other species. On the other hand, sediment resuspension might only slightly impact the community composition in the water column when germination is largely driven by seasonality and the internal clock. The aim of this study was, therefore, to explore the impact of sediment resuspension on microbial eukaryote community composition in the water column of a shallow coastal ecosystem in the context of seasonal species succession, focusing on all unicellular eukaryotes, which we also refer to as protists. To investigate this, we compared the unicellular eukaryotic community composition in the supernatants of undisturbed and regularly mixed sediment cores from Greifswald Bay over 4 months, while water temperature increased from 5 to 20 °C. The core supernatants were siphoned off every few weeks, the protist community was collected on filters for metabarcoding of the 18S rRNA genes, and 0.2-μm filtered seawater was carefully poured back onto the sediment cores.

## 2 Materials and methods

### 2.1 Sampling

Six sediment cores (inner diameter 10 cm) were collected using a multicorer device (Oktopus, Kiel, Germany) from Greifswald Bay (lat: 54.23233, long: 13.55017) on 13 March 2024 for an incubation experiment at a location with a water depth of 8.4 m. Each core was approximately 60% filled with sediment and contained approximately 2 L of supernatant water. At the same time, additional sediment cores were collected for porewater nutrient analysis. Porewater was extracted using Rhizons (pore size: 0.12–0.14 μm, Rhizosphere, Wageningen, Netherlands) down to a depth of 30 cm (*n* = 3). Sampling was conducted at 1-cm intervals for the upper 10 cm and at 4-cm intervals below this depth. One additional sample was taken out of the supernatant water. The samples were stored at −20 °C until further analysis. Ten liters of surface water were also collected to assess the *in situ* microbial community composition. This water was consecutively filtered through 5-μm and 0.2-μm membrane filters to collect microplankton separately from nanoplankton and picoplankton. The filters were stored at −80 °C until further processing, and the filtered water was kept at 4 °C.

### 2.2 Determination of OPD

To determine the oxygen penetration depth (OPD), sediment cores (inner diameter: 2.1 cm) were subsampled from larger cores and transported to the laboratory while maintaining near *in situ* temperatures. OPD measurements were conducted using an oxygen microelectrode (OX-100, Unisense, Aarhus, Denmark), which was connected to a microsensor multimeter and a microprofiling system (both Unisense, Aarhus, Denmark). The electrode was polarized 1 h prior to measurements and two-point calibrated immediately before use. To capture the benthic boundary layer, the electrode was placed ~5 mm above the sediment. OPD was measured over 10 cycles with data recorded at 200-μm intervals using the micro profiling system.

### 2.3 Experimental set-up

All six sediment cores were incubated in parallel for approximately 4 months (mid-March to early July) in temperature-regulated chambers, where the surrounding cooling water followed the *in situ* temperature of Greifswald Bay. At the beginning of the experiment, the water temperature in Greifswald Bay was approximately 5 °C, and in early July, it reached 20 °C. Three of them served as controls, while the other three were regularly mixed. To ensure stable temperatures and to mimic light conditions at the bottom of Greifswald Bay, which has a median secchi depth of 1.6 m (water monitoring data, Landesamt für Umwelt, Naturschutz und Geologie Mecklenburg-Vorpommern), and to avoid excessive benthic growth, the experiment was performed in the dark. The supernatant in each core was continuously bubbled to prevent anoxic conditions. One day after setting up the experiment (T0), the supernatant was siphoned from every sediment core. A few milliliters of water remained on the sediment to avoid further resuspension and sampling of benthic organisms. The supernatant was consecutively filtered through a 55-μm mesh to remove zooplankton, a filter membrane of 5 μm pore size to collect microplankton, and a 0.2-μm membrane filter for nanoplankton and picoplankton. The filters were stored at −80 °C until further processing. Afterward, the core was immediately refilled with temperature-adjusted, sterile-filtered seawater from Greifswald Bay to enable future identification of microorganisms that exclusively originate from the sediment. In three randomly chosen cores, the upper 1.5 cm of sediment was resuspended with an oscillating disk (8 cm diameter) that was slowly moved up and down in the uppermost 15 cm of the water column on days 11 (T1), 47 (T2), 61 (T3), 89 (T4), and 110 (T5) of the experiment. The other three cores represented the control treatment without mixing. One to five days before each mixing event, the supernatant from every sediment core was siphoned off and filtered as described above. The cores were refilled with temperature-adjusted, sterile-filtered seawater from the previous sampling of the same core. Two days after each mixing event when most of the resuspended sediment had settled again, the planktonic microbial communities were again collected and the cores re-filled (Supplementary Figure 1). At the end of the experiment, sediment samples were taken at depths of 5 mm, 10 mm, 15 mm, 20 mm, and 30 mm in each core to assess the diversity of potential resting stages.

### 2.4 Nutrient analysis

During each sampling event, 10 mL of 0.2-μm filtered water from every core was stored at −20 °C for nutrient analysis. Nutrients in supernatant water from the experiment and porewater were measured colorimetrically according to Grasshoff et al. (2009) by means of a Seal Analytical QuAAtro automated constant flow analyzer. The influence of experimental treatment (mixing vs. control) and timing of sampling (pre- vs. post-mixing) on nutrient concentrations in the supernatant was analyzed using a repeated-measure ANOVA in R version 4.4.1 with mixing event (T1–T5) as an error stratum.

### 2.5 Molecular analysis

DNA was extracted from all water samples using the MagMax Waste Water Treatment kit (Applied Biosystems, Waltham, Massachusetts, United States) and from all sediment samples using the Qiagen Power Soil pro kit (Qiagen, Hilden, Germany). To analyze the eukaryotic community composition, the V4 region of the 18S rRNA gene was amplified using the primers TAReuk454FWD1 and TAReukREV3 (Stoeck et al., 2010). The library was paired-end sequenced on an Illumina Nova PE250 platform.

The raw sequencing data were processed using an in-house developed snakemake pipeline (Hassenrueck, 2022), which includes primer clipping, the exclusion of read-pairs lacking the expected primer sequences located at the 5′ ends of the reads, and the elimination of read-pairs that have misplaced primer sequences. DADA2 (Callahan et al., 2016) was used for denoising, allowing at most two errors (maxEE) in the forward reads and at most three errors in the reverse reads, as well as a minimum fragment length of 225 bp, concatenating paired-end reads, and chimera removal. The resulting 18S rRNA amplicon sequence variants (ASVs) were taxonomically assigned with DADA2 using PR2 version 5.0.0 (Guillou et al., 2013) as a training set for 18S rRNA gene amplicons.

### 2.6 Statistical analyses

The eukaryotic community composition was further analyzed using R version 4.4.1 packages “phyloseq” (McMurdie and Holmes, 2013) and “vegan” (Oksanen et al., 2016). As this study focuses on unicellular eukaryotes, Metazoa and Streptophyta ASVs were removed from the dataset, and heterotrophic bacteria and zooplankton were also not further discussed. The read depth of all samples was rarified to 51,678 reads. Eukaryotic beta diversity was visualized using barplots and Venn diagrams. Alpha diversity indices (observed richness, and Shannon and Simpson diversity) were calculated and plotted using the R package “phyloseq.” The influence of treatments (control vs. mixing) and timing of sampling (pre- vs. post-mixing) on alpha diversity was investigated with a repeated-measure ANOVA, taking mixing events (T1–T5) into account as an error stratum. With a canonical correspondence analysis (CCA), using the R package vegan, the impact of treatments (control vs. mixing), size fractionation (0.2–5 μm vs. 5–55 μm), timing of sampling (pre- vs. post-mixing), temperature, and nutrient concentrations (PO_3_^−^, NO_3_^−^, NO_2_^−^, NH_4_^+^, and SiO_2_) on eukaryotic community composition were analyzed. All ASVs were clustered, when possible, on the genus level, but some taxa could only be assigned to higher taxonomic groups. The continuous variables were normalized by standardizing their mean to 0 and standard deviation to 1 before the analysis. The best model was selected based on automatic stepwise model building using the function ordistep in the R package “vegan”, starting with a model that included only the intercept and adding all variables afterward. Only genera that belong to the 10% most abundant genera and to the 30% of genera with the smallest distance to variable centroids were selected with the ordiselect function from the R package “goeveg” for displaying in the CCA plot. Additionally, the impact of the treatments, size fractionation (0.2–5 μm vs. 5–55 μm), timing of sampling, temperature, and nutrient concentrations on Bray-Curtis distances between samples with ASVs merged at the genus level were investigated with a repeated measure PERMANOVA with date as blocks for the permutations using the adonis2 function in the R package “vegan”. Temperature data and nutrient concentrations were again normalized before the PERMANOVA analysis. Pairwise comparisons between treatments, size fractions, and timing of sampling were performed with the R package “pairwiseAdonis” (Arbizu, 2020). The impact of treatment and sampling horizon on the eukaryotic community composition in the sediment was also analyzed using a PERMANOVA. A differential abundance analysis was performed using the R package DESeq2 to identify genera that were differentially abundant in core supernatant samples from mixed and control treatments. Only genera with more than 1% relative read abundance were included in the analysis, and *p*-values were adjusted using Benjamini and Hochberg method (Benjamini and Hochberg, 1995).

## 3 Results

Between 1,401,292 and 51,678 eukaryotic reads remained per sample after filtering, merging of forward and reverse reads, and removal of ASV affiliated with Metazoa and Streptophyta (Supplementary Table 1). The sediment samples had, on average, the highest read depth but lost a high proportion (~15%) of reads during the removal of metazoans. After rarefaction, the remaining sequences were assigned to 16,405 different eukaryotic ASVs belonging to 1,220 different genera, although taxonomic assignment was not always possible on this level.

**Figure 1:**
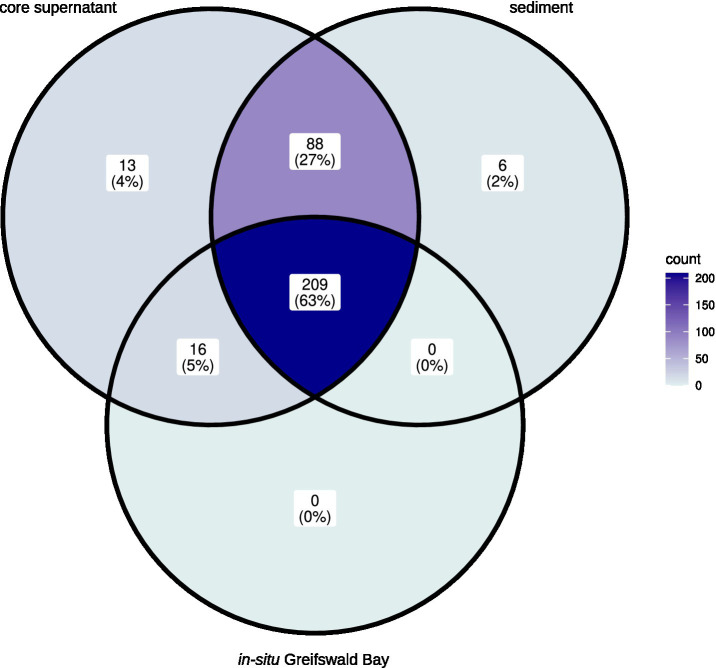
Venn diagram showing the presence and absence of the 332 most abundant genera (> 0.01% relative read abundance) in core supernatant, sediment, and *in-situ* Greifswald Bay samples.

The majority (63%) of the most abundant eukaryotic genera (>0.01% relative read abundance) occurred in all three types of samples, namely in the *in-situ* water samples from Greifswald Bay, the core supernatants from the experiments, and the sediment samples. However, the water samples from Greifswald Bay were less diverse than the core supernatant and sediment samples (Figure 1). Besides the 225 eukaryotic genera that were also found in Greifswald Bay water samples, 88 additional genera hatched from the sediment and were found in the core supernatant. Additionally, 13 taxa, when possible taxonomically assigned at the genus level, were uniquely found in the core supernants, namely an unclassified Gregarinidae (Apicomplexa), Holophryidae (ciliate), Ochromonadales_clade-XII_X, the Radiozoa *Amphibelone*, unclassified Acantharea_F and unclassified Acantharea_2 (Radiolaria), the dinoflagellate *Karlodinium*, the ciliate *Parauronema*, NPK2-lineage_X (Cercozoa), Spumellaria_6_X (Radiozoa), the ciliate *Pseudocohnilembus*, the Cercozoa *Thaumatomonas,* and the Ascomycota *Candida.* Among these, the ciliate genus *Parauronema* had the highest relative read abundance and mainly occurred in one core in the pre- and post-mixing samples from T2, but also in very low relative abundances at T0. Surprisingly, very few genera uniquely occurred in the sediment, namely an amoebae from the Breviata-lineage, the Cercozoa *Neoheteromita*, the diatom *Actinocyclus*, an unclassified Litostomatea (ciliate), the green alga *Chlorococcum*, and the Chrysophyceae *Naegeliella*.

### 3.1 Sediment characteristics

At the beginning of the experiment, the silty sediment was anoxic below 4.4 mm depth when mean oxygen concentrations dropped below 10 μmol L^−1^ (Supplementary Figure 2). Mean NH_4_^+^ concentrations increased from 1.5 μmol L^−1^ in the water just above the sediment to 253 μmol L^−1^ in the porewater at 3.5 cm sediment depth. Below this horizon, NH_4_^+^ concentrations slightly decreased again before increasing further to 316 μmol L^−1^ at 25.5 cm depth (Supplementary Figure 3). In the water just above the sediment, on average, we measured 23 μmol L^−1^ of nitrate, but its concentrations in the porewater quickly decreased and it was depleted at 1.5 cm sediment depth at T0. Similarly, mean NO_2_^−^ concentrations reached 0.2 μmol L^−1^ in the water above the sediment and were not detectable anymore below 1.5 cm sediment depth. Mean PO_4_^−^ concentrations continuously increased from 0.07 μmol L^−1^ in the water above the sediment to almost 60 μmol L^−1^ in porewater at 33.5 cm sediment depth, while mean SiO_2_ concentrations increased from 19 μmol L^−1^ to 480 μmol L^−1^ (Supplementary Figure 3).

At the end of the experiment, the sediment samples were in general dominated by reads from the eustigmatophyte *Monodus*, the diatom *Skeletonema*, the chlorophytes *Choricystis* and *Picochlorum,* and the genus *Sphaeropleales*, the dinoflagellate *Pelagodinium*, a parasite of the Syndiniales Group I Clade 4, a genus of the Apicomplexa family Lecudinidae, and an unclassified dinoflagellate (Supplementary Figure 4). A PERMANOVA on the sediment samples indicated significant differences (R^2^ = 0.236, *p* = 0.001) in the protist community composition between different sediment sampling horizons, as well as weak, non-significant differences (R^2^ = 0.05, *p* = 0.055) between mixed and control cores. A pairwise PERMANOVA showed significant differences (*p* < 0.01) in community composition between the surface sediment samples (H05 = 5 mm depth) and the deeper horizons (H15, H20, and H30: 15–30 mm depth). When focusing on the upper sediment horizons (H05, H10, and H15: 5–15 mm depth) that were regularly resuspended during the experiment, both the treatment and the sampling horizon had a significant impact on protist community composition (R^2^_treatment_ = 0.12, p_treatment_ = 0.026; R^2^_horizon_ = 0.2, p_horizon_ = 0.023).

### 3.2 Protist diversity in core supernatants

**Figure 2:**
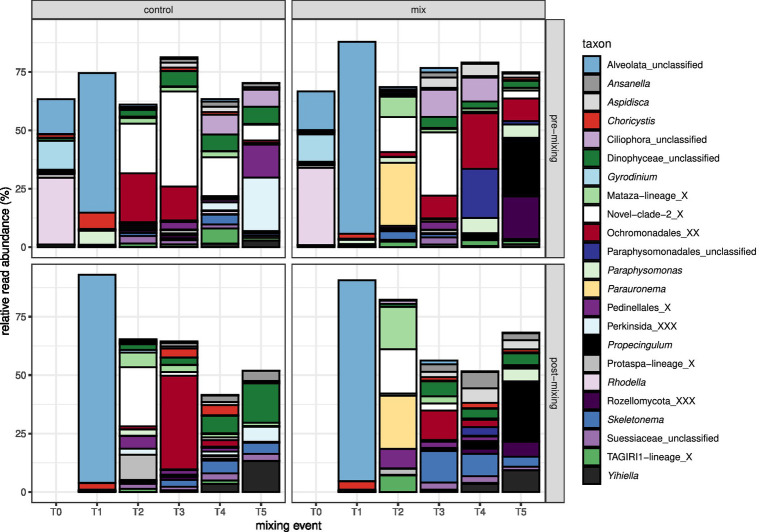
Barplot showing the relative read abundance of the most abundant protist taxa (>1% relative read abundance), clustered at the genus level, in the 5–55 μm fraction of the core supernatants.

Protist cells continuously moved from all sediment cores (mixed and control treatments) into the supernatant throughout the experiment, showing a notable temporal progression in community composition over time in both size fractions. At T0, 1 day after setting up the experiment and before any mixing, the protist community composition in the 5–55 μm size fraction was in all core supernatants, characterized by reads from the unicellular red algae *Rhodella*, the dinoflagellate *Gyrodinium,* and unclassified Alveolata (Figure 2). Approximately 1 week later (T1), just before and after the first mixing event, the core supernatants were completely dominated by the unclassified Alveolata. The closest, taxonomically classified hit of this ASV based on a BLASTn search on the NCBI nucleotide database was the parasitic Alveolata species *Perkinsus olseni* with 59% query coverage and 95% sequence identity. The 0.2–5 μm size fraction was on T0 as well as on T1, dominated by reads of the potentially parasitic unclassified Alveolata.

**Figure 3:**
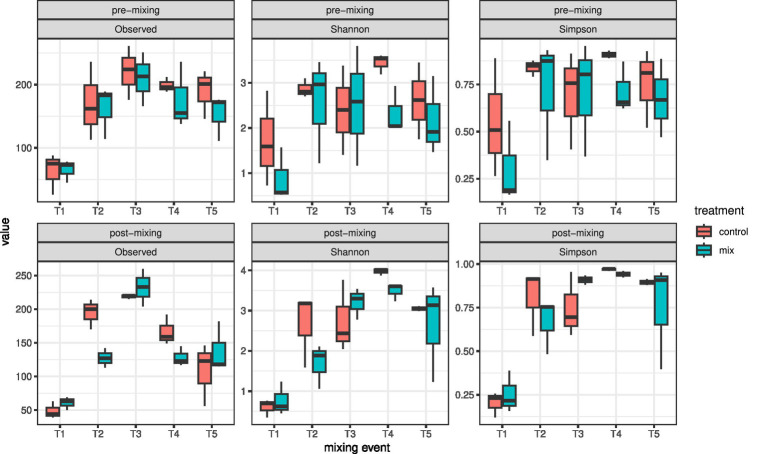
Boxplots showing trends in alpha diversity indices of the 5–55 μm size fraction across mixing events during the experiment.

From T2 on (approximately one month later), the alpha diversity increased in both size fractions and treatments. The observed alpha diversity peaked at T3 and decreased afterwards again, while Shannon and Simpson diversity remained relatively stable from T3 to the end of the experiment (Figure 3). Overall, the observed alpha diversity was significantly higher in the pre-mixing than in the post-mixing communities (repeated measures ANOVA *p* = 0.049), but there was no significant difference between the two treatments. In contrast, Shannon diversity was slightly higher in the control cores than in the mixed cores (repeated measures ANOVA *p* = 0.0684). The Simpson diversity did not differ between treatments or timing of sampling.

From T2 onward, Ochromonadales, two Cercozoa genera (Novel-Clade-2 and Mataza-lineage), the unicellular red algae *Rhodella*, the planktonic diatom *Skeletonema*, the ciliates *Propecingulum* (Spirochtrichea) and *Parauronema* (Oligohymenophorea), an unclassified Dinophyceae, and Pedinellales (silicoflagellates) were the genera with the highest relative read abundance in the 5–55 μm fraction (Figure 2). In the 0.2–5 μm fraction, Ochromonadales, Perkinsida, Novel-Clade-2 from the Cercozoa, the chlorophyte *Choricystis*, the stramenopile MAST-12A, Pedinellales (silicoflagellates), the fungal group Rozellomycota, and an unclassified Dinophyceae displayed the highest relative read abundances (Supplementary Figure 5).

### 3.3 Impact of experimental treatments and environmental factors on protist community composition

**Figure 4:**
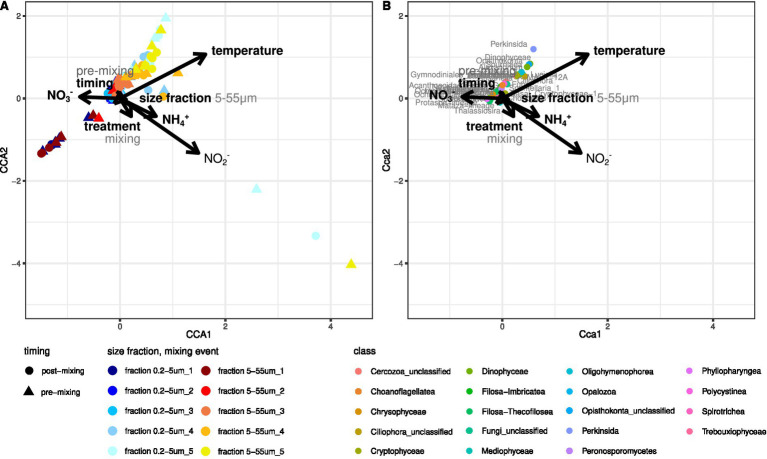
**(A)** CCA illustrating the ordination of core supernatant samples (CCA site scores) based on experimental treatments, sampling, and nutrient concentrations. **(B)** CCA illustrating the impact of experimental treatments, sampling, and nutrient concentrations on eukaryotic community composition (CCA species scores). Only protists that belong to the 10% most abundant genera and to the 30% of genera with the smallest distance to variable centroids are plotted. All plotted factors were statistically significant in the CCA, bold factors were also statistically significant in a repeated-measure PERMANOVA.

Multiple factors varied throughout the experiment and likely impacted the protist community composition in core supernatant samples. Temperature and SiO_2_ and PO_3_^−^ concentrations were correlated and significantly increased throughout the experiment (Temperature: 5–20 °C, SiO_2_: 62–294 μmol L^−1^, PO_3_^−^: 0.4–4.5 μmol L^−1^) (Figure 4), while NO_3_^−^ and NH_4_^+^ concentrations significantly differed between the supernatants of the mixed sediment core and control treatments (ANOVA: p_NO3_ = 0.026, p_NH4_ = 0.033). NO_3_^−^ concentrations were higher in the control treatments (mean = 30.8 μmol L^−1^, sd = 10.6 μmol L^−1^ vs. mean = 23.7 μmol L^−1^, sd = 12 μmol L^−1^), while NH_4_^+^ concentrations were higher in the supernatants of the mixed sediment cores (mean = 3.9 μmol L^−1^, sd = 1.6 μmol L^−1^ vs. mean = 15.2 μmol L^−1^, sd = 26.1 μmol L^−1^) (Supplementary Figure 6). A repeated measure PERMANOVA indicated significant effects (*p* < 0.05) of experimental treatment (control vs. mixing), timing of sampling (pre- vs. post-mixing), size fractionation during sampling (0.2–5 μm vs. 5–55 μm), temperature, NO_3_^−^ concentrations, and NH_4_^+^ concentrations on the eukaryotic community composition in the core supernatants (Supplementary Table 2A). Based on the corresponding F and R^2^ values, temperature and size fractionation accounted for most of the variation in the dataset, whereas experimental treatment contributed the least. A canonical correspondence analysis (CCA) supported these findings and suggested a significant impact of temperature, NO_2_^−^ concentrations, NH_4_^+^ concentrations, NO_3_^−^ concentrations, size fractionation, timing of sampling, and experimental treatment on protist community composition in core supernatant samples (Figure 4). According to the CCA, the included environmental variables explain 23.9% of the inertia in the community dataset, thus the majority of the variance remained unexplained.

When splitting the dataset into pre- and post-mixing samples, the impact of these factors on community composition changed according to repeated measure PERMANOVA results (Supplementary Tables 2B,C). In the post-mixing samples, the protist community composition did not differ between the control and mixed cores. However, there was still a significant impact from temperature, size fractionation, and NO_3_^−^ concentrations on community composition. Additionally, PO_3_^−^ and NO_2_^−^ concentrations significantly impacted the protist community composition in the post-mixing samples. In contrast, in the pre-mixing samples, only the experimental treatment, size fractionation, and NO_3_^−^ and NO_2_^−^ concentrations remained significant. There was no significant effect of temperature on protist community composition in core supernatant samples anymore.

**Figure 5:**
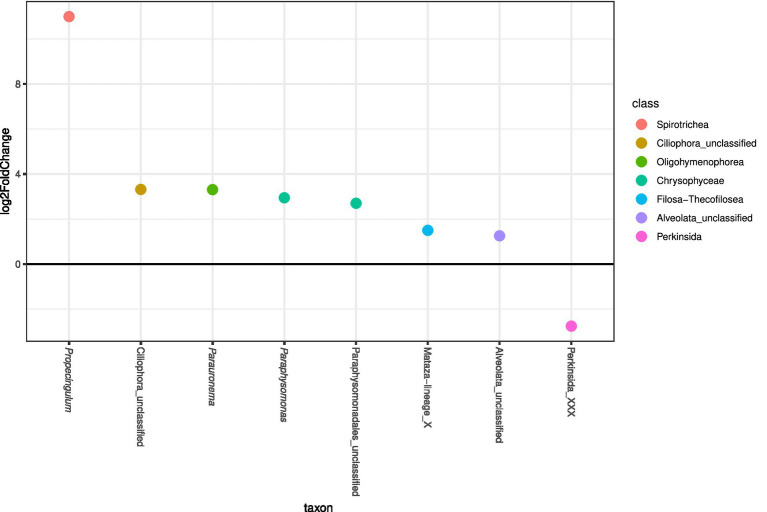
Differential abundance analysis of most abundant protist taxa (>0.01% relative read abundance), clustered at the genus level, in mixed and controlled treatments of the core supernatant. Taxa with > 0 log2FoldChange are more abundant in the supernatant from mixed cores, while taxa with < 0 log2FoldChange are more abundant in the supernatant from control cores.

A Perkinsida, a MAST-12A genus, the dinoflagellate genera *Sourniaea*, *Alexandrium*, *Luciella*, *Heterocapsa,* and *Ansanella*, as well as some unclassified Dinophyceae, Opisthokonta, and Ciliophora were most strongly affected by the rise in temperature over time and primarily appeared in the core supernatants toward the end of the experiment (Figure 4B). According to the differential abundance analysis, several genera were significantly more abundant in the mixed cores than in the control treatments (Figure 5), in particular the ciliates *Propecingulum* and *Parauronema*, two genera from the chrysophyte order Paraphysomonadales, and a Cercozoa from the Mataza-lineage. In contrast, a Perkinsida genus occurred more in the control cores.

## 4 Discussion

To predict planktonic protist community dynamics under climate change, an understanding of processes affecting life stage transitions, including recruitment of cells from the sediment, is important. Resuspension of sediment and thereby transport of previously buried eukaryotic resting stages into the oxygenated water column might facilitate germination and proliferation of different species. In this study, the planktonic community composition noticeably changed due to recruitment from the sediment. Few protist taxa uniquely occurred in the sediment, although parts of the benthic diversity, in particular Metamonads or Euglenozoa, might be poorly resolved with the V4 region of the 18S rRNA gene (Choi and Park, 2020; Novák et al., 2024). Instead, the majority of taxa also appeared in the water column belonging to groups, such as chrysophyceae (Holen and Princiotta, 2023), Cercozoa (Dumack et al., 2016, 2017), diatoms (Ellegaard and Ribeiro, 2017), ciliates (Li et al., 2022), and Pedinellales (Daugbjerg, 1996), which are known to germinate from resting stages under favorable conditions. Some Cercozoa and ciliates in the water column might also represent benthic taxa that were resuspended through mixing. However, as sampling took place as early as 2 days after the mixing events, we expect that most resuspended resting stages, which did not germinate due to unfavourable environmental conditions, and benthic species re-settled to the sediment and were not collected during filtration of the core supernatants. A similar decrease of seston and benthic ciliates in the water column of an incubation experiment was observed by Garstecki et al. (2002) after 48 h post-mixing. However, benthic rhizopods and diatoms remained in the water column of their experiment for a longer time period.

At the first mixing event, the eukaryotic community in both size fractions was dominated by an unclassified alveolate parasite that was likely carried over in a few remaining milliliters of supernatant from T0 (Figure 2). As the entire planktonic community was removed by filtration 2 days after mixing, the plankton in the following samples was likely recruited from the sediment to the water column. This eukaryotic pelagic community changed over time and differed between regularly mixed and control sediment cores. Many heterotrophic and mixotrophic taxa were detected in the water column, and phototrophic phytoplankton species successfully germinated despite the lack of light during our experiment. These findings are supported by another laboratory experiment (Hedberg et al., 2024), which showed that light was not essential for the germination of several phytoplankton groups. Instead, diatoms best germinated under cold and dark conditions, while germination of cyanobacteria and dinoflagellates primarily increased with higher temperatures in spring but not with illumination.

Sediment resuspension had a small but significant impact on community composition in the water column as well as in the top 1.5 cm of the sediment. Many protist species are able to leave the surface of the sediment and enter the water column with the help of flagella, gas vesicles, or positive buoyancy (e.g., Richardson et al., 1996). However, as soon as the cells are buried by sediment, independent movement into the water column is very limited. Furthermore, anoxic conditions, a few millimeters under the sediment surface (Supplementary Figure 2), likely inhibit germination. In this experiment, certain ciliate, cercozoan, and chrysophyte taxa benefited from resuspension, transporting these previously buried organisms into the oxygenated water column and facilitating their germination. Although the species richness did not differ between mixed and control treatments, the Shannon diversity indices were slightly lower in the mixed treatment, indicating dominance of some species potentially due to their increased germination rates.

Mixing was, however, not the primary factor in our experiment. The recruitment was largely impacted by temperature and NO_3_^−^ concentrations in the water column (Figure 4). Temperature is one of the most important environmental factors regulating survival and reproduction in plankton as well as causing shifts in cell numbers and community composition over time (Agrawal, 2009). A shift in temperature is the primary stimulus for excystment in many protist species and usually drives seasonal community succession, as every species has its own temperature window and tolerance limit for germination (Anderson and Morel, 1979; Gyllström and Hansson, 2004; Ellegaard and Ribeiro, 2017). For example, the dinoflagellate genera *Sourniaea*, *Alexandrium*, *Luciella*, *Heterocapsa,* and *Ansanella*, as well as a Perkinsida genus, were only recruited to the water column toward the end of our experiment when water temperature exceeded 12 °C and 16 °C, respectively (Figure 4B). The strong effect of temperature in this experiment supports, thus, a generally large contribution of germination to the recruitment of cells from the sediment.

In our experiment, resuspension resulted in decreased nitrate and elevated ammonium concentrations in the mixed treatment compared to the control treatment (Supplementary Figure 6). The effects of nitrate and ammonium concentrations on germination of resting stages and protist growth are complex and differ between species. In some dinoflagellate species, elevated ammonium concentrations induced encystment within a few days in laboratory experiments (Rao and Stewart, 2011; Wang et al., 2014) and inhibited germination from akinetes in the cyanobacterium *Nodularia spumigena* (Huber, 1985). On the other hand, many phytoplankton species prefer using ammonium over nitrate due to its more efficient transport over the cell membrane, which can, in turn, repress nitrate assimilation (Glibert et al., 2016). Furthermore, a lack of nitrogen as well as excessive nitrogen concentrations can decrease hatching rates from spores and cysts in some microalgae (Rengefors and Anderson, 1998; Agrawal, 2009). Nitrate concentrations in the experiment were likely impacted by variable uptake rates of the protist community over time and between the treatments. In the mixed treatment, benthic ciliates and diatoms could partly be responsible for the removal of nitrate from the water column. During temporary resuspension, these organisms might intracellularly store nitrate for assimilatory nitrate reduction (DNRA) in returning anoxic conditions (Kamp et al., 2015). Furthermore, mixing probably transported nitrate from the water column into the sediment. Previous studies showed similarly increased removal of NO_3_^−^ from the water due to elevated denitrification rates in silty sediments in the dark (Sundbäck et al., 2000). Resuspension of the sediment also released accumulated ammonium from the sediment into the supernatant in the mixed treatments (Garstecki et al., 2002; Lawrence et al., 2004).

The timing of sampling also had a significant impact on the observed eukaryotic community composition in the core supernatants. The post-mixing sampling was scheduled 2 days after the resuspension events to allow resettling of sediments, detect initiated germination, and avoid die-off of phototrophic species due to the lack of light. However, other experiments showed a start of germination of several phytoplankton species and a following increase in cell concentrations between 2 and 7 days after the onset of favorable conditions (Nehring, 1996; Rengefors and Anderson, 1998; Hedberg et al., 2024). Thus, the post-mixing sampling probably took place too early to detect a significant impact of the sediment resuspension on eukaryotic community composition in the water column. Instead, the community composition in the post-mixing samples was strongly impacted by gradually changing environmental parameter, in particular temperature and to a lesser extent nutrient concentrations such as PO_3_^−^, NO_3_^−^, NO_2_^−^, and NH_4_^−^concentrations (Supplementary Table 2B). In contrast, the “pre-mixing” samples, 2 weeks to 1 month after the previous mixing events, recorded a stronger impact of the experimental treatment (Supplementary Table 2C). After the mixing event, several previously buried resting stages probably resettled to the surface of the sediment. As germination was initiated some days after the on-set of favorable, aerobic conditions, the vegetative cells only moved after the post-mixing sampling into the water column. The growth of these initially resuspended cells, some potentially activated outside of their usual growth season, was, thus, only detected in the following pre-mixing samples temporarily overshadowing the signal of seasonal succession.

Light limitation during the experiment likely reduced the growth of phototrophic taxa. Although phototrophic eukaryotes, such as the chlorophyte genera *Desmodesmus* and *Picochlorum*, as well as the Ochrophyta *Monodus*, *Nannochloropsis,* and *Thalassiosira*, were highly abundant in the sediment (Supplementary Figure 4), mainly heterotrophic and mixotrophic taxa, such as Cercozoa and ciliates occurred in higher relative read abundances in the water column. Many phototrophic taxa likely germinated in the dark as sunlight does not reach the sea floor of Greifswald Bay (median secchi depth = 1.6 m, Landesamt für Umwelt, Naturschutz und Geologie Mecklenburg-Vorpommern) and were detected in low relative read abundance in the core supernatants. Their vegetative growth was, however, limited during the experiment. Nevertheless, the planktonic diatom *Skeletonema marinoi* appeared in higher relative read abundance in the middle of May, when the water temperature reached approximately 12 °C. This temperature is close to the described temperature optimum of *Skeletonema marinoi* (Hattich et al., 2024), suggesting that this typical spring-bloom diatom species continuously germinates from the sediment and could grow to noticeable cell concentrations at higher temperatures when competition is missing.

In general, this experiment highlighted the large influence of temperature-dependent germination from resting stages in the sediment on the eukaryotic plankton community composition. The growth season and succession in the plankton might, thus, shift in the future, when the timing of germination is changed due to climate warming (Hallegraeff, 2010). In particular, dinoflagellate genera, such as *Sourniaea*, *Alexandrium*, *Luciella*, *Heterocapsa,* and *Ansanella,* which were notably favored by higher temperatures in this experiment, might germinate earlier under current climate change scenarios. Hedberg et al. (2024) also demonstrated increased cell abundances in this group in experimental treatments with higher temperature. Similarly, Jerney et al. (2019) observed faster germination under elevated temperature in the dinoflagellate *Alexandrium ostenfeldii*, although the overall germination success in this species was not affected by temperature. Furthermore, sediment resuspension by storms provides an inoculum of new cells to the water column, which can multiply and change the planktonic community composition if environmental parameters are suitable for their growth. This effect was noticeable in different plankton communities that naturally succeeded each other over the course of a few months in this experiment. Associated changes in nutrient concentrations in the water column through benthic–pelagic coupling will further affect planktonic community composition.

## Data Availability

The data presented in the study are deposited in the European Nucleotide Archive (ENA), accession number PRJEB97091.
