# Supplementary material for: The impact of temperature and sediment resuspension on microbial eukaryote recruitment from shallow Baltic Sea sediment
Source: Front Microbiol. 2025 Nov 21;16:1654505. doi: 10.3389/fmicb.2025.1654505 (PMC12679096; doi:10.3389/fmicb.2025.1654505)
Supplement: Supplementary file 1 [file Supplementary_file_1.pdf]

Supplementary materials

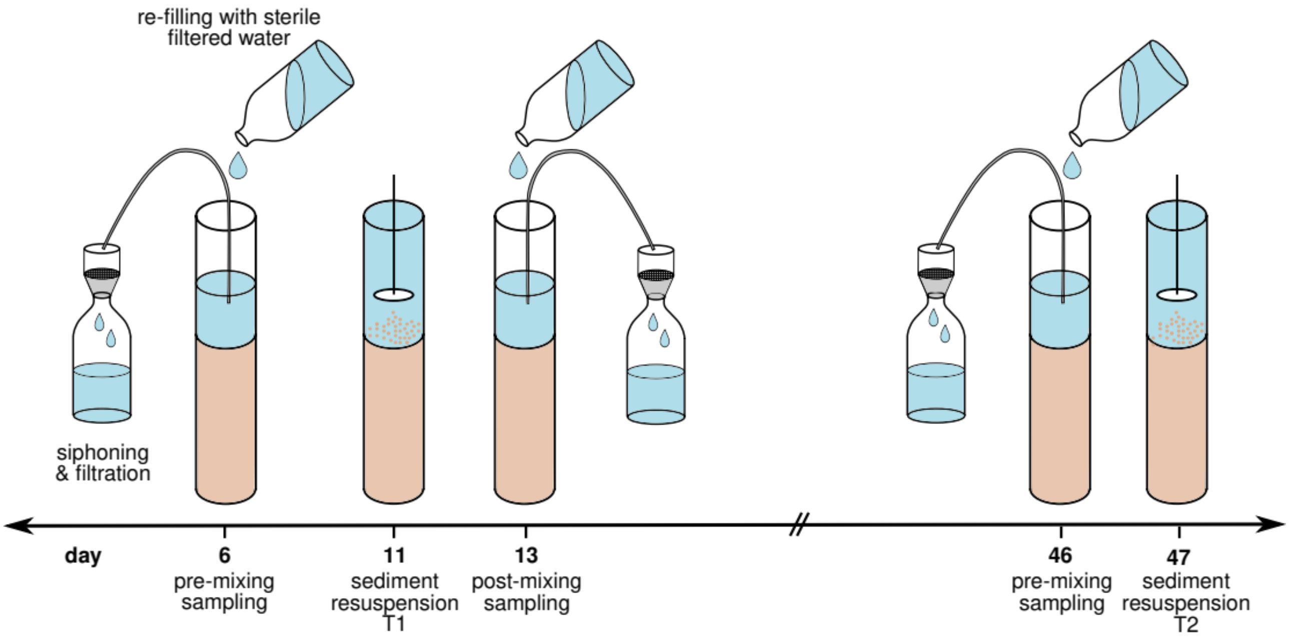

Figure 1: Example of temporal arrangement of sampling around the mixing events during the experiment.

Table 1: Mean number of reads from core supernatant, sediment and Greifswald Bay samples kept during processing in data2 and after removal of metazoans.

**Processing step**	**core supernatant**	**sediment**	**Greifswald Bay**
Demux	287447.9	613994.4	304444.3
Clipped_fr	281646.8	604171.2	298969.3
Filtered_fr	260157.8	550683.5	276424.3
Denoised_fwd_fr	259586.2	549113.1	275726.0
Denoised_rev_fr	259726.9	548724.9	275634.3
Merged_fr	252877.1	532607.0	270827.0
Nochim	244565.3	490156.2	246456.0
Tabled	244551.7	490073.1	246416.6
remove_metazoa	227189.8	419025.7	224171.7

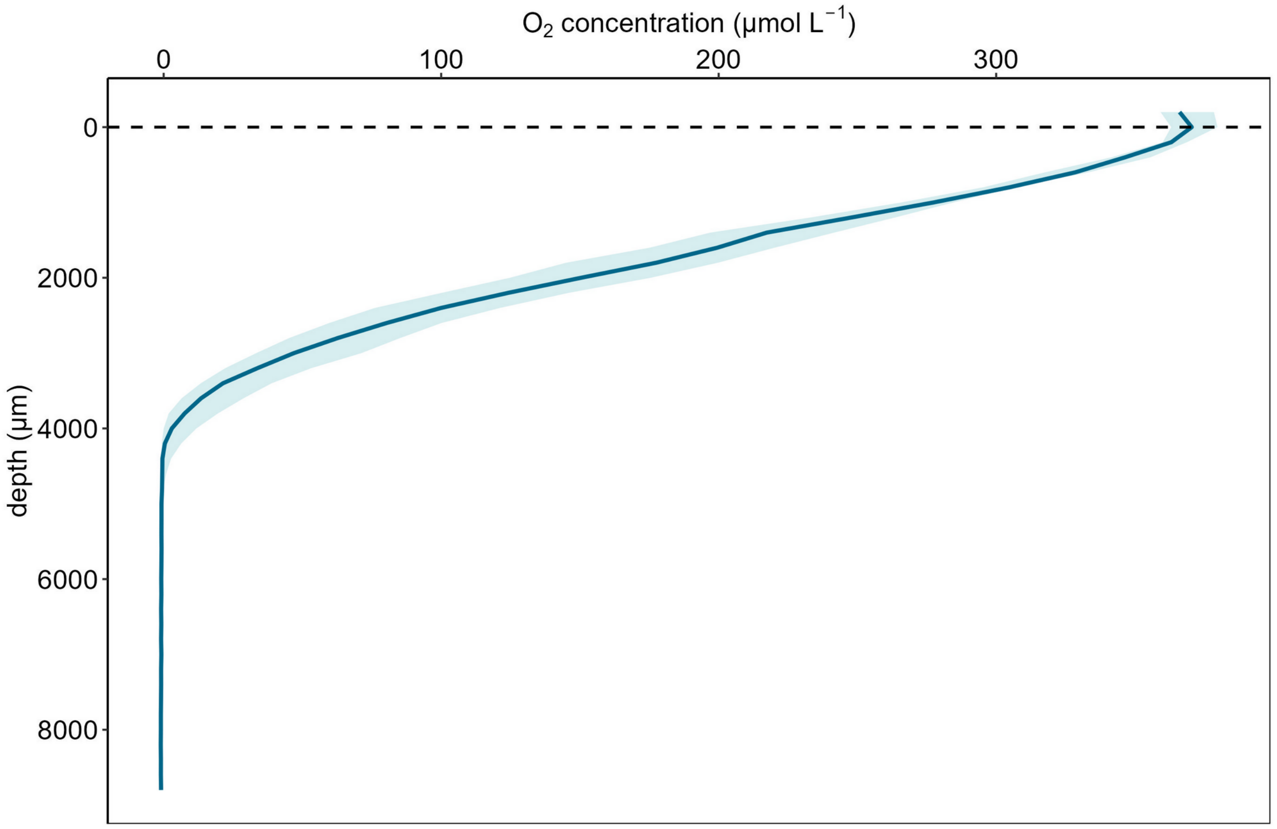

Figure 2: Depth profile of median oxygen concentration in porewater with interquartile range (0.25 and 0.75; n= 10) at T0. The dashed line indicates the sediment water interface.

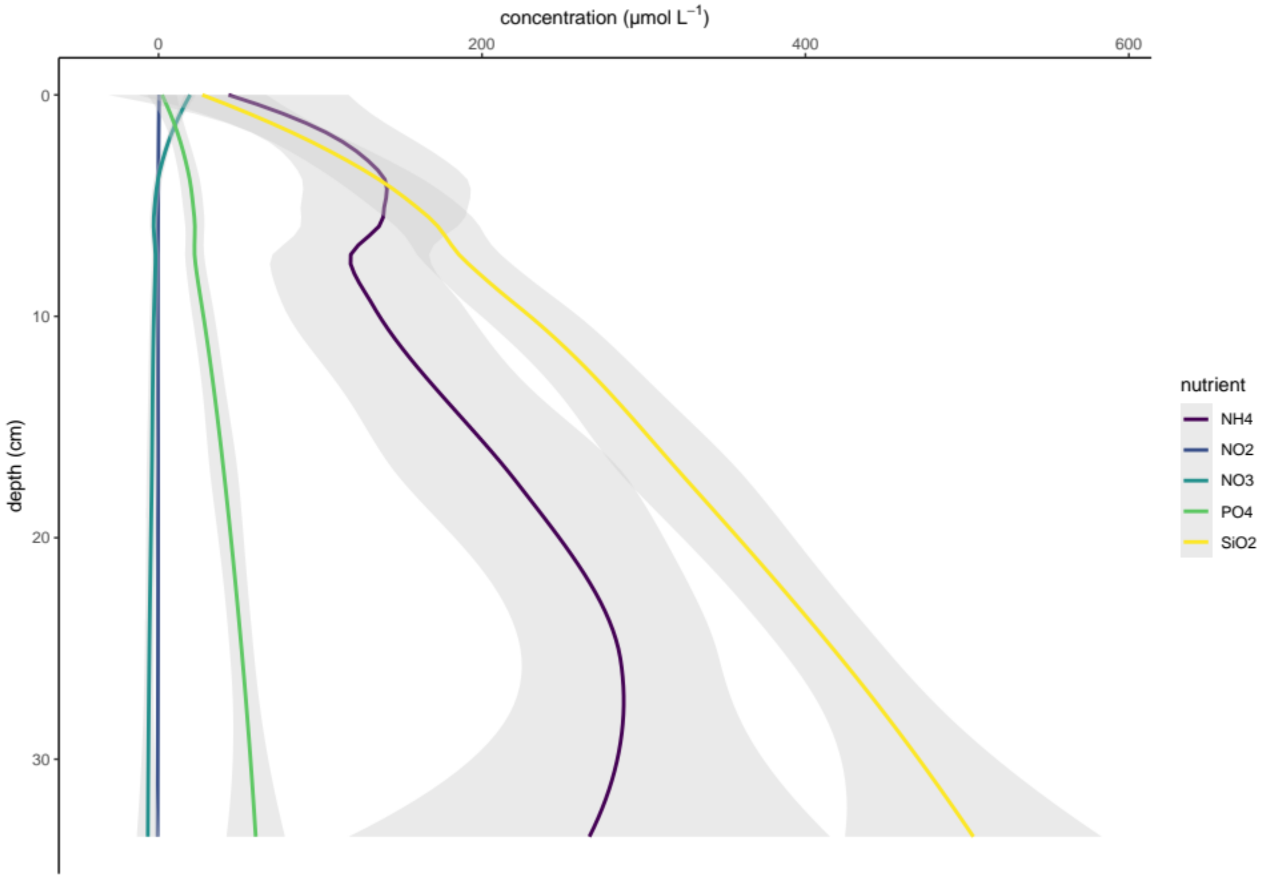

Figure 3: Nutrient concentration profiles in porewater from three sediment cores at T0.

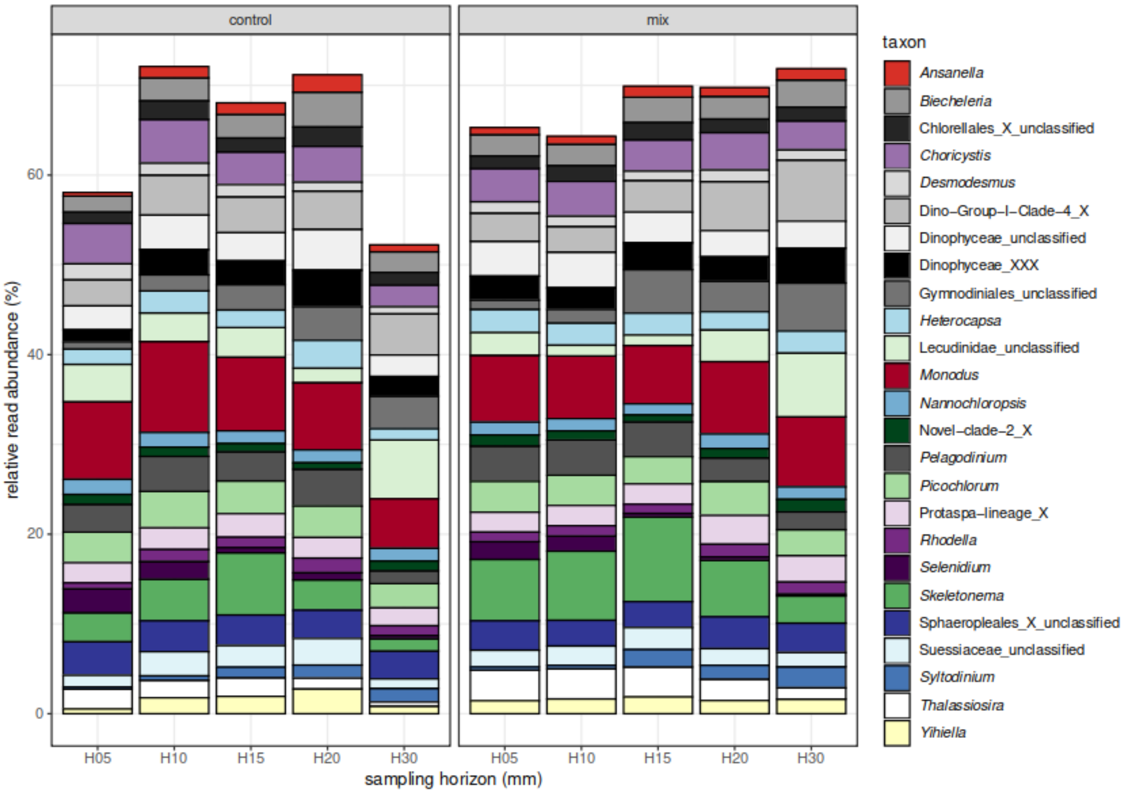

Figure 4: Barplot showing the relative read abundance of the most abundant protist taxa (>1% abundance), clustered at the genus level, in different sediment layers at the end of the experiment.

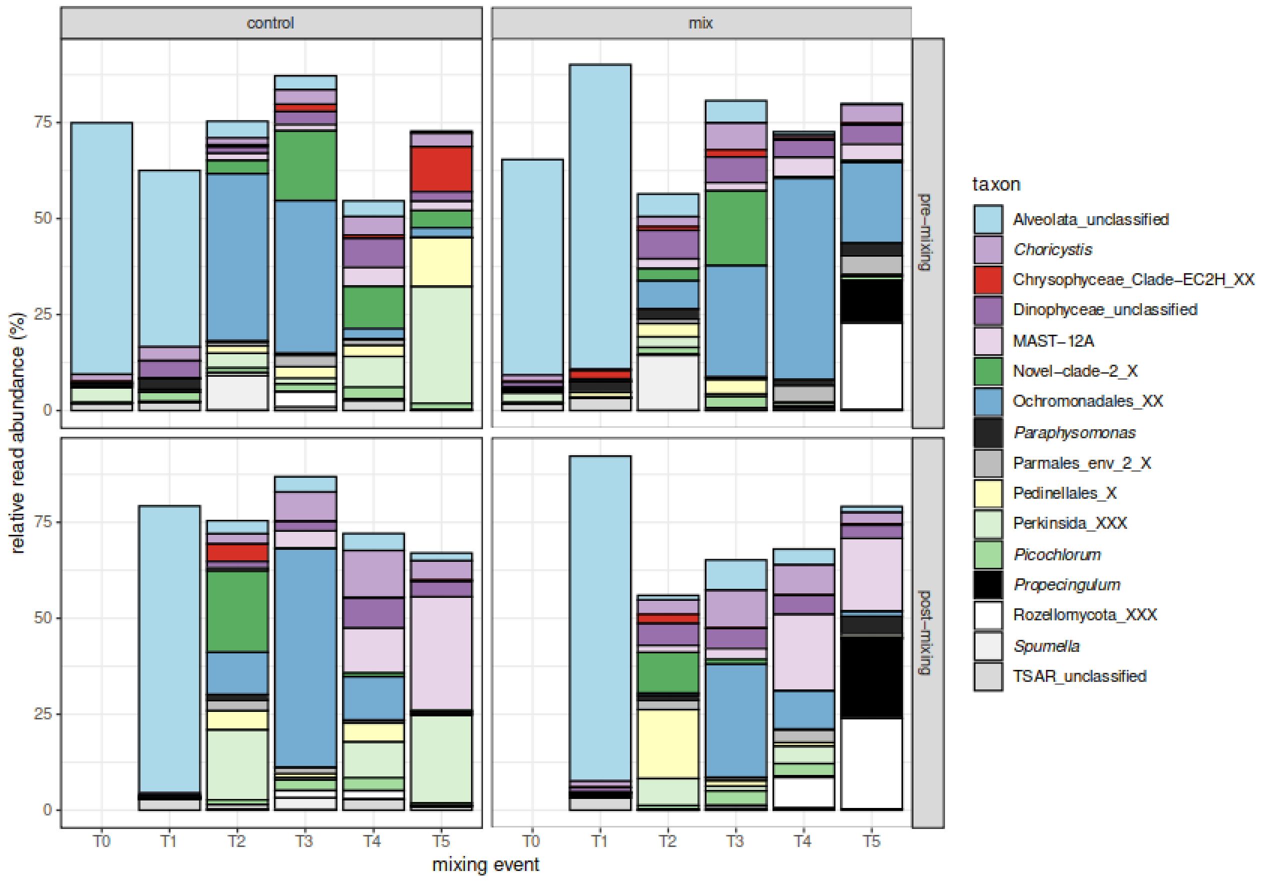

Figure 5: Barplot showing the relative read abundance of the most abundant protist taxa (>1% relative read abundance), clustered at the genus level, in the 0.2-5 µm fraction of the core supernatants.

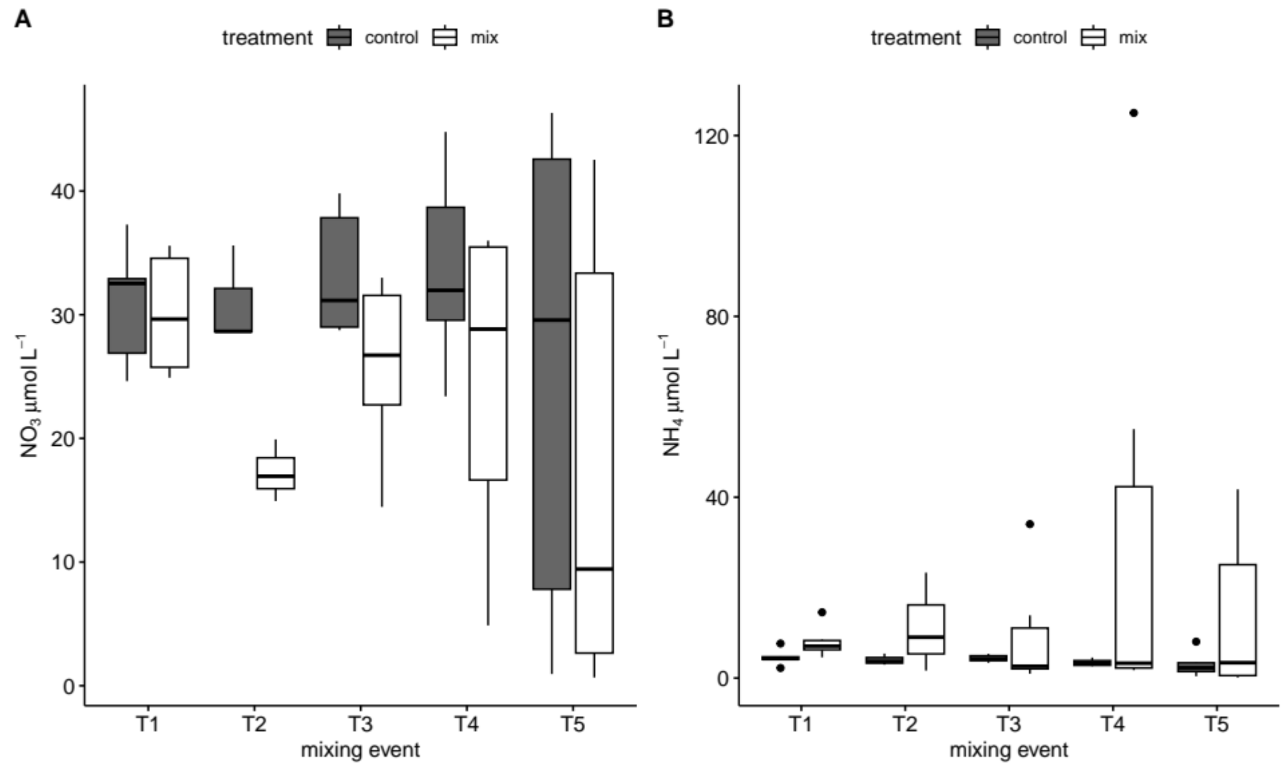

Figure 6: NO_3^- and NH_4^+ concentrations in the core supernatants across mixing events during the experiment.

Table 2: Results from repeated measure PERMANOVAs based on Bray-Curtis distances between samples with protist ASVs merged at the species level for A) the entire data set, B) only post-mixing samples and C) only pre-mixing samples.

**A) all data**	**Df**	**SumOfSqs**	**R2**	**F**	**Pr(>F)**
treatment	1	0.400	0.011	1.733	0.009 **
size fraction	1	1.792	0.049	7.768	0.001 ***
temperature	1	1.143	0.031	4.955	0.003 **
PO_4^-	1	3.204	0.087	13.893	0.003 **
NO_3^-	1	0.726	0.020	3.148	0.219
NO_2^-	1	1.184	0.032	5.133	0.001 ***
NH_4^+	1	0.564	0.015	2.444	0.184
SiO_2	1	0.894	0.024	3.877	0.008 **
Residual	1	0.304	0.008	1.319	812.000
Total	98	22.602	0.617		
treatment	107	36.655	1.000		

**B) post-mixing**	**Df**	**SumOfSqs**	**R2**	**F**	**Pr(>F)**
treatment	1	0.189	0.013	1.145	0.357
size fraction	1	1.143	0.078	6.918	0.001 ***
temperature	1	2.280	0.155	13.798	0.001 ***
PO_4^-	1	0.467	0.032	2.824	0.014 *
NO_3^-	1	0.461	0.031	2.789	0.016 *
NO_2^-	1	0.581	0.040	3.516	0.001 ***
NH_4^+	1	0.395	0.027	2.389	0.035 *
SiO_2	1	0.275	0.019	1.666	0.324
Residual	37	6.113	0.416		
Total	45	14.682	1.000		

**C) pre-mixing**	**Df**	**SumOfSqs**	**R2**	**F**	**Pr(>F)**
treatment	1	0.479	0.025	1.897	0.008 **
size fraction	1	0.972	0.051	3.847	0.001 ***
temperature	1	0.591	0.031	2.338	0.830
PO_4^-	1	0.434	0.023	1.717	0.321
NO_3^-	1	0.595	0.031	2.353	0.008 **
NO_2^-	1	0.562	0.030	2.224	0.009 **
NH_4^+	1	0.449	0.024	1.776	0.089 .
SiO_2	1	0.299	0.016	1.182	0.383
Residual	49	12.384	0.651		
Total	57	19.028	1.000		
